# Efficacy and toxicities of doxorubicin plus ifosfamide in the second-line treatment of uterine leiomyosarcoma

**DOI:** 10.3389/fonc.2023.1282596

**Published:** 2023-11-28

**Authors:** Szu-Yun Niu, Lou Sun, Shih-Tien Hsu, Sheau-Feng Hwang, Chih-Ku Liu, Yu-Hsiang Shih, Ting-Fang Lu, Yen-Fu Chen, Li-Ching Lai, Pei-Lun Chang, Chien-Hsing Lu

**Affiliations:** ^1^ Department of Obstetrics and Gynecology, Taichung Veterans General Hospital, Taichung, Taiwan; ^2^ Department of Food and Nutrition, Providence University, Taichung, Taiwan; ^3^ Center for General Education, Ling Tung University, Taichung, Taiwan; ^4^ School of Medicine, China Medical University, Taichung, Taiwan; ^5^ Department of Public Health, Chung Shan Medical University, Taichung, Taiwan; ^6^ Ph.D. Program in Translational Medicine, and Rong Hsing Research Center for Translational Medicine, National Chung Hsing University, Taichung, Taiwan

**Keywords:** uterine leiomyosarcoma, second-line treatment, doxorubicin plus ifosfamide, gemcitabine plus docetaxel, adjuvant chemotherapy

## Abstract

**Purpose:**

Uterine leiomyosarcoma is a rare and aggressive tumor known for its drug resistance and metastatic potential. The standard first-line treatment typically involves anthracycline-based chemotherapy or a combination of gemcitabine and docetaxel; however, there is currently no established second-line treatment. Therefore, the aim of this study was to evaluate the efficacy and toxicity of doxorubicin plus ifosfamide as a potential second-line treatment for uterine leiomyosarcoma.

**Materials and methods:**

This is a retrospective, single-center, single-arm study. We reviewed the tumor registry data from January 2010 to December 2022 and identified patients with uterine leiomyosarcoma who had previously received first-line salvage or adjuvant treatment involving gemcitabine and taxotere, and later experienced tumor recurrence. Patients who met these criteria were included in the study. The primary endpoint was the efficacy of doxorubicin and ifosfamide as a second-line treatment for uterine leiomyosarcoma, as measured by progression-free survival, 1-year overall survival, and response rate. The secondary endpoint was the adverse events associated with this regimen.

**Results:**

Fifty-two patients were diagnosed with uterine leiomyosarcoma during the study period, nine of whom were included in the data analysis. All patients had previously received gemcitabine-docetaxel as first-line adjuvant therapy, with a median progression-free survival period of 8.4 months. Doxorubicin-ifosfamide was administered as second-line treatment, with a median progression-free survival of 6.0 months (range: 2.7-79.9 months). The clinical benefit rate of the second-line treatment was 66.7%, with a median overall survival of 33.0 months, and a 1-year overall survival rate of 83.3%. Previous reports have shown that the median progression-free survival for second-line treatments using other regimens ranged from 1.4-5.6 months. The most common adverse event was myelosuppression, with five patients requiring granulocyte colony-stimulating factor and one patient requiring a blood transfusion. No patient discontinued treatment due to unmanageable adverse events.

**Conclusion:**

Use of doxorubicin with ifosfamide may be a promising and reasonable second-line treatment with manageable adverse events for patients with uterine leiomyosarcoma.

## Introduction

Uterine leiomyosarcoma is a rare malignancy arising from the myometrium, accounting for only 1-2% of uterine malignancies (1–3). Despite its low incidence, it behaves aggressively and has poor prognosis, with a reported recurrence risk of 50-71% after hysterectomy (4–6). The 5-year survival rate for patients with stage I to IV disease range from 22% to 55% (1, 7). Diagnosis of uterine leiomyosarcoma is based on histologic examination, with the three most important criteria being mitotic index, cellular atypia, and geographic areas of coagulative necrosis separated from viable neoplasm (8). However, preoperative diagnosis of uterine leiomyosarcoma can be challenging, with ultrasound having a diagnostic sensitivity of only 11% and often being unable to differentiate the condition from uterine leiomyoma (9).

Based on the 2023 NCCN guidelines, the recommended initial treatment for uterine leiomyosarcoma is total hysterectomy with or without bilateral salpingo-oophorectomy. For patients who are not qualified candidates for primary surgery, systemic therapy or palliative radiation therapy should be considered. Adjuvant systemic therapy should also be considered for patients who are diagnosed with stage II to IV disease after primary surgery. The anthracycline-based regimen, such as doxorubicin, has been the mainstay of first-line adjuvant treatment since 1985, with a response rate of 30.3% (10–13). The median progression-free survival associated with doxorubicin single-use ranges from 4.6-5.8 months (11–16).

Ifosfamide, a DNA alkylating agent used in cancer chemotherapy, has predominantly been studied in combination therapy (17). Nonetheless, when ifosfamide was used as a single agent, a previous study demonstrated a median progression-free survival of 3.8 months and a partial response in 17.2% of patients (18). A randomized controlled Phase 3 trial, published in 2014, compared the efficacy of doxorubicin alone versus the combination of doxorubicin and ifosfamide as first-line treatment for advanced or metastatic soft-tissue sarcoma. The study demonstrated a significantly longer median progression-free survival in the doxorubicin with ifosfamide group (7.4 months) compared to the doxorubicin group (4.6 months) (19). Consequently, our study will focus on the doxorubicin and ifosfamide regimen as second-line treatment. Gemcitabine-docetaxel is another first-line adjuvant treatment option, which was first recognized as a treatment option in 2002 and has a response rate of 35.7–53.0%, with a median progression-free survival period of 4.4–13.0 months (20–22).

Recurrence within a year is common in patients with advanced uterine leiomyosarcoma, and standard second-line therapy has not yet been established (23). Trabectedin is a preferred regimen for patients who have received prior anthracycline-containing treatment, with a reported progression-free survival period of 5.4 months (24). Previous studies have demonstrated the efficacy of several treatment options, including gemcitabine, gemcitabine-docetaxel, ixabepilone, etoposide, dacarbazine, trabectedin, eribulin, paclitaxel-carboplatin, trimetrexate, ifosfamide, doxorubicin and doxorubicin-dacarbazine, in second-line or later treatments, for which the median progression-free survival period ranges from 1.4-5.6 months (22, 23, 25–37).

Currently, there is limited research on the use of doxorubicin-ifosfamide as second-line treatment for uterine leiomyosarcoma, particularly in patients with prior exposure to gemcitabine-docetaxel. Therefore, in this study, we aim to evaluate the treatment efficacy and adverse effects of doxorubicin-ifosfamide combination therapy in patients with uterine leiomyosarcoma who have previously received treatment with gemcitabine-docetaxel.

## Methods

This retrospective, single-center, single-arm study was conducted at Taichung Veterans General Hospital and received approval from the Institutional Review Board. Data from the tumor registry at the hospital, covering the period from January 2010 to December 2022, were reviewed. The pathological staging of the patients was determined based on the International Federation of Gynecology and Obstetrics 2009 classification.

### Patient selection

All patients included in the study had either undergone prior incomplete resection procedures, such as laparoscopic myomectomy, followed by subsequent complete resection surgery, involving total hysterectomy with or without bilateral salpingo-oophorectomy, or had received complete resection as their primary treatment. Following surgical intervention, all the patients received first-line adjuvant therapy with gemcitabine-docetaxel. Upon the detection of disease recurrence or metastasis as confirmed by a computerized tomography (CT) scan, second-line adjuvant or salvage therapy involving doxorubicin-ifosfamide was administered. Only the histology type of leiomyosarcoma was included in the study, while other types, such as carcinosarcoma, adenosarcoma, endometrial stromal sarcoma, and undifferentiated uterine sarcoma, were excluded. Interval debulking surgery and radiotherapy prior to second-line chemotherapy were permitted.

### Procedures and monitoring protocol

After the confirmation of disease progression or recurrence through abdominal computed tomography, participants received second-line chemotherapy treatment consisting of doxorubicin 30-40 mg/m^2^ on day 1 intravenously over 2 hours and ifosfamide 1.2 Gm/m^2^ from days 1-3 over a period of 72 hours, along with mesna 1.2 Gm/m^2^ from days 1-3 over 72 hours. Chemotherapy was administered every 3 weeks until unacceptable side effects or disease progression occurred. Doxorubicin was stopped while the cumulative dose was approximated to 450mg/m^2^ in the patient’s lifetime due to concerns of cardiac toxicity (38).

Blood chemistry and urine analysis were obtained prior to each cycle of chemotherapy. The side effects were assessed according to the International Common Toxicity Criteria (version 2.0). Abdominal CT scans were performed every 3 months to evaluate the location, number, and size of the tumors. The patient’s disease status was assessed using the RECIST 1.1 criteria. The primary endpoints of this study were progression-free survival, 1-year overall survival, and response rate. The secondary endpoint was the assessment of treatment-related side effects.

Statistical analyses were performed using SPSS version 22.0 (IBM Corp., Armonk, NY, USA). Progression-free survival was analyzed using the Kaplan-Meier method.

## Results

### Patient characteristics

**Figure 1:**
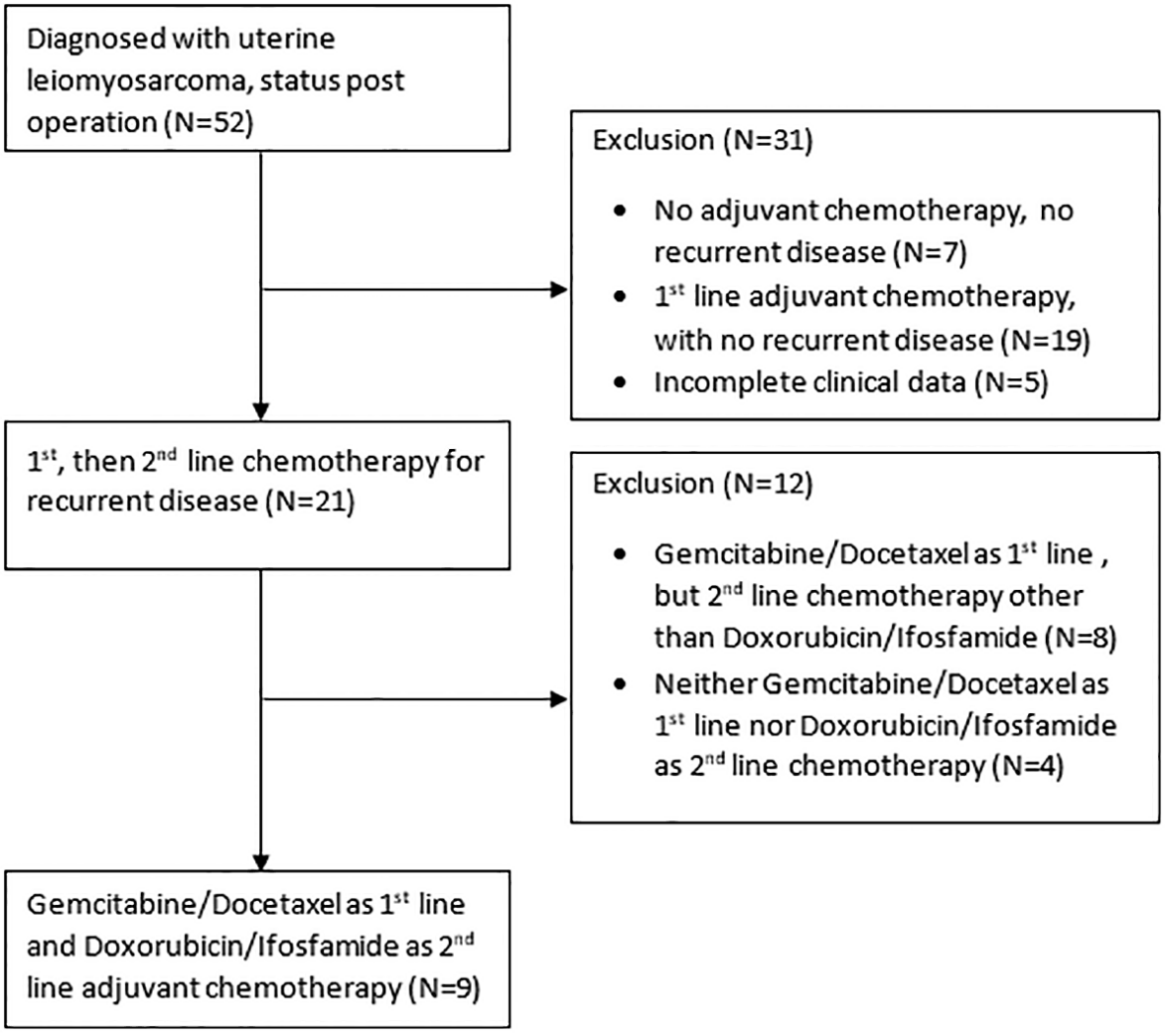
Flow diagram of the patient selection.

Nine females were included in the study (**Figure 1**), with ages ranging from 44-66 years (median age: 55 years). Among them, eight patients were diagnosed with early-stage disease (stage I or II), while only one patient had reached advanced-stage disease at diagnosis. Three patients received incomplete resection at first, followed by complete resection. Six had received complete hysterectomy as their primary treatment. All patients received first-line adjuvant chemotherapy cycles of gemcitabine-docetaxel, with a median number of four cycles. One patient had received radiotherapy prior to the study (**Table 1**).

**Table 1:** Patients characteristics (n=9).

Characteristic	Number of patients
Age at diagnosis	
<40	0
40-49	2
50-59	5
≥60	2
FIGO stage	
Stage I, II	8
Stage III, IV	1
Surgery	
Myomectomy preceding hysterectomy	3
Hysterectomy with or without BSO as primary treatment	6
Cycle numbers of first-line Gemcitabine/Docetaxel adjuvant chemotherapy	
2	1
3	0
4	4
5	2
6	1
≥7	1

All patients experienced disease recurrence or metastasis following first-line adjuvant therapy. The metastatic sites included the bones, liver, gastrointestinal tract, and lungs. The largest metastatic tumor, located on the left abdomen, measured 17.2 cm in diameter. The median size of metastatic tumors was 5.4 cm in diameter. One patient underwent video-assisted thoracic surgery for the resection of lung tumors, achieving R0 status before receiving second-line adjuvant therapy with doxorubicin-ifosfamide. The remaining eight patients received second-line doxorubicin-ifosfamide chemotherapy as salvage treatment. Three patients (33%) received six or more cycles of doxorubicin-ifosfamide. The median number of doxorubicin-ifosfamide cycles administered was five. The maximum number of cycles received by a patient was 14, after which, she discontinued treatment due to the accumulated doxorubicin dose nearing 450mg/m^2^, and subsequently switched to doxorubicin hydrochloride liposome (**Table 2**).

**Table 2:** Disease status and treatment at recurrence.

Disease status and treatment at recurrence	Number of patients
Radiotherapy after recurrence before Doxorubicin/Ifosfamide treatments	1
Disease status at the starting of second-line treatments	
R0 disease	1
R1 disease	0
R2 disease	8
Sites of metastasis at the starting of second-line treatments	
Bone	1
Liver	1
Gastrointestinal tracts	1
Lung	4
Multiple metastasis	2
Cycle numbers of second-line Doxorubicin/Ifosfamide adjuvant chemotherapy	
4	2
5	4
6	1
7	0
≥8	2

### Response to treatment and survival

**Figure 2:**
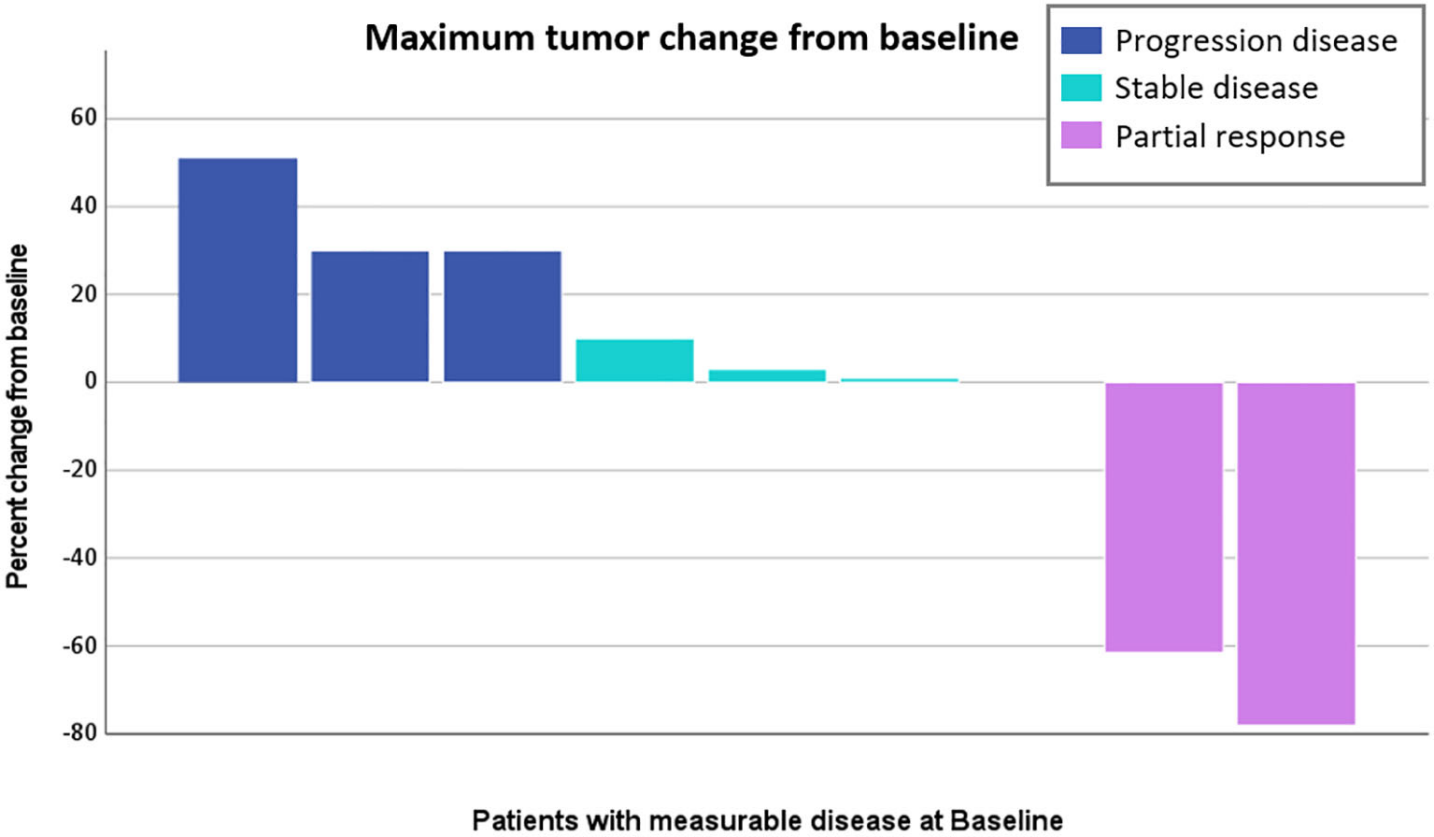
Maximum tumor change from baseline of second-line doxorubicin/ifosfamide treatments.

In this cohort study of nine patients experiencing recurrence, the median progression-free survival for the first-line adjuvant therapy of gemcitabine-docetaxel was 8.4 months. In the second-line treatment, no complete response was observed in these nine patients. A partial response was observed in two patients (22.2%), with response durations of 24.2 and 8.4 months, respectively. The overall response rate was 22.2%. Four patients (44.4%) had stable disease, with progression-free survival of 79.9, 9.8, 6.0, and 2.8 months, respectively. The clinical benefit rate was 66.7%. Three patients (33.3%) experienced disease progression, with median progression-free survival of 2.8 months (**Table 3**, **Figure 2**).

**Table 3:** RECIST-defined response to treatments of second-line doxorubicin/ifosfamide treatments.

Response category	Number of patients	Percentage
Complete response	0	0
Partial response	2	22.2
Stable disease	4	44.4
Progression of disease	3	33.3
Total	9	100

**Figure 3:**
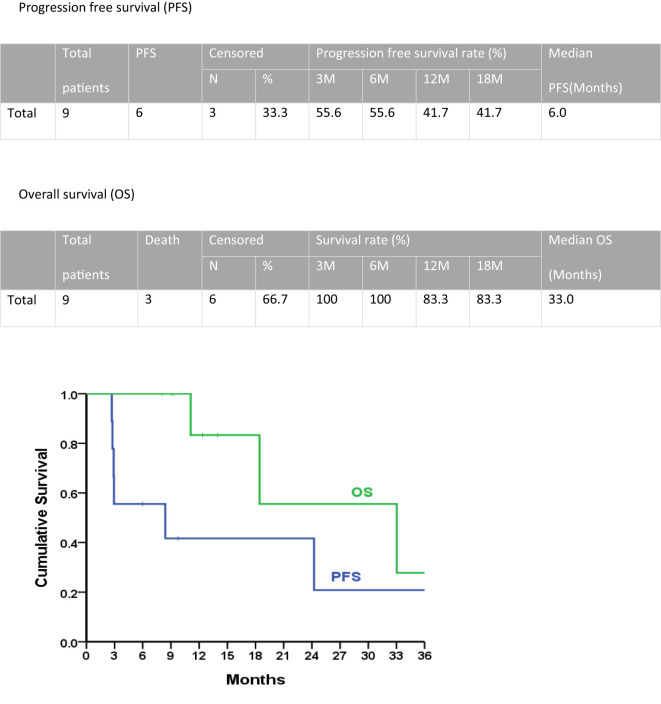
Progression-free survival and overall survival of second-line doxorubicin/ifosfamide treatments.

The median progression-free survival period for all patients in the second-line treatment was 6.0 months, with a 1-year progression-free survival rate of 41.7% (**Figure 3**). The median overall survival was 33.0 months, and the 1-year overall survival rate was 83.3%.

### Adverse events

A summary of the adverse events is demonstrated in **Table 4**. The most common toxicity was myelosuppression, with four patients experiencing grade 1-2 neutropenia, and two patients experiencing grade 3-4 neutropenia. One patient had grade 1-2 leukopenia, while five had grade 3-4 leukopenia. Grade 1-2 anemia was observed in four patients. Granulocyte colony-stimulating factor was administered to five patients (56%), and one patient (11%) received a blood transfusion. Mild elevation in liver function tests was noted in two patients, while three patients experienced grade 1-2 renal toxicity. No patients exhibited central nervous system toxicity and no patient discontinued the doxorubicin and ifosfamide treatment due to intolerable adverse events.

**Table 4:** Adverse events of second-line doxorubicin/ifosfamide treatment.

Adverse events	Grade 1-2	Grade 3-4
Neutropenia	4(44%)	2(22%)
Leukopenia	1(11%)	5(56%)
Anemia	4(44%)	0(0%)
Thrombocytopenia	0(0%)	0(0%)
Liver toxicity	2(22%)	0(0%)
Renal toxicity	3(33%)	0(0%)
Central nervous system toxicity	0(0%)	0(0%)

## Discussion

In this cohort study of nine patients experiencing recurrence, patients with uterine leiomyosarcoma received gemcitabine-docetaxel as first-line adjuvant chemotherapy. The median progression-free survival with this first-line treatment was 8.4 months. Doxorubicin-ifosfamide was administered as a second-line treatment. This second-line treatment approach demonstrated a median progression-free survival period of 6.0 months, with a notable 1-year progression-free survival rate of 41.7%. Furthermore, the median overall survival was 33.0 months, while the 1-year overall survival rate reached 83.3%.

Anthracycline-based chemotherapy has been the standard first-line adjuvant treatment for sarcomas during the past four decades. However, the gemcitabine-docetaxel combination emerged as an alternative option in 2002 (22). Despite these advancements, a standard second-line treatment for leiomyosarcomas has not yet been established.

Previous studies have explored the efficacy of different treatment options for uterine leiomyosarcoma. A study published by Sutton et al. in 1996 demonstrated a median response duration of 4 months with doxorubicin and ifosfamide in first-line treatment (11). However, this promising result was challenged by the study published by Hensley et al. in 2002 which showed a median progression-free survival of 5.6 months with gemcitabine-docetaxel in first and second-line settings (22). The encouraging result of gemcitabine-docetaxel was also shown in the study by Seddon et al. in 2015 which revealed a median progression-free survival period of 7.1 months in the first-line treatment of unresectable leiomyosarcoma (39).

In 2017, the GeDDiS trial compared the treatment efficacy of doxorubicin and gemcitabine-docetaxel in previously untreated advanced unresectable or metastatic soft tissue sarcoma. The results showed no significant difference in progression-free survival between the two treatment groups in first-line treatment (23.3 weeks [95% confidence interval: 19.6–30.4] vs. 23.7 weeks) (40). However, the GeDDiS trial and the study conducted by Hensley and colleagues in 2002 show some differences, including the histology type (soft tissue sarcoma vs. uterine leiomyosarcoma), gemcitabine dose (675 vs. 900 mg/m2), and cycles (6 vs. 6–8 cycles). A recent study published by Pautier et al. in 2022 showed an encouraging result coming from the doxorubicin and trabectedin combination as a first-line treatment in soft tissue leiomyosarcomas and uterine leiomyosarcomas. The patients in that study experienced a median progression-free survival period of 12.2 months in the doxorubicin and trabectedin combination group and 6.2 months in the doxorubicin alone group (41). Most studies investigating doxorubicin and ifosfamide included various histology types of soft tissue sarcoma, but we speculate that the gemcitabine-docetaxel combination may yield better responses, particularly in patients with uterine leiomyosarcoma.

In second-line studies, the combination of gemcitabine and docetaxel has shown promising treatment efficacy in uterine leiomyosarcoma. Hensley et al. demonstrated a median progression-free survival of more than 5.6 months with this combination (23). Similarly, a cohort study by Pautier et al. reported a median progression-free survival of 4.7 months upon using the gemcitabine and docetaxel combination (28). Other treatments, such as ixabepilone and etoposide, as second-line options for uterine leiomyosarcoma, showed a median progression-free survival of 1.4 and 2.1 months, respectively (22, 23, 25–27). In addition to these treatments, other regimens have been used as treatments beyond the second line, including gemcitabine-dacarbazine, dacarbazine, trabectedin and eribulin. However, these regimens have demonstrated inferior treatment effects, with median progression-free survival ranging from 2.6 to 4.9 months (32–34).

Herein, we focused on patients who had received gemcitabine and docetaxel as a first-line treatment before receiving the doxorubicin and ifosfamide combination as a second-line therapy. The dosage of doxorubicin in our study (30-40 mg/m^2^) was lower than in previous studies, where dosages of 50-60 mg/m^2^ were commonly used in the combination regimen (50mg/m^2^ in Sutton et al. combined with ifosfamide (11), 60mg/m^2^ in Pautier et al. combined with trabectedin). Our decision to use a lower dosage was based on our experience when treating Asian patients, as higher doses of doxorubicin tended to cause intolerable side effects. We found that if the patient had advanced disease, we could administer more than six cycles of doxorubicin and ifosfamide, if the accumulative dosage did not exceed 450 mg/m^2^, so as to avoid cardiotoxicity (38). Despite using a lower dosage, the median progression-free survival period was still 6.0 months, suggesting that the lower dosage did not result in inferior treatment efficacy. The most common toxicity observed was myelosuppression, but no patients discontinued the study due to intolerable side effects. We managed the side effects by administering granulocyte colony-stimulating factor and blood transfusions to sustain further treatment cycles.

This study’s limitation are the small number of patients, as we only included nine individuals and the lacking control group. Following a review of the literature, we also found recent studies on biomarker-directed therapy in both first and second-line treatments. Information on biomarkers such as the NTRK gene, tumor mutational burden, microsatellite status, and BRCA mutation holds significance. In these nine patients, we identified two patients who underwent Foundation One genetic testing, both of whom exhibited microsatellite stability and low tumor mutational burden, with no subsequent biomarker-directed therapy administered (42, 43). The absence of genetic testing in certain cases can be attributed to various factors, including high costs of testing and targeting drugs, rare instances of positive findings, or the early era of diagnosis when genetic testing was not widely employed, while, the effectiveness of targeted therapy had not been fully studied. Despite these limitations, we demonstrated a median progression-free survival of 6.0 months with doxorubicin-ifosfamide as second-line treatment in these nine patients. This finding provides a potential treatment option for second-line therapy in patients with uterine leiomyosarcoma and highlights the need for further research involving larger sample sizes.

In conclusion, our study present a treatment strategy for patients with uterine leiomyosarcoma, which involved the use of gemcitabine-docetaxel as a first-line adjuvant chemotherapy following primary complete surgery, and doxorubicin-ifosfamide as second-line treatment. This treatment approach demonstrated promising results in terms of both progression-free survival and overall survival, with patients experiencing manageable side effects.

## Data availability statement

The original contributions presented in the study are included in the article/supplementary material. Further inquiries can be directed to the corresponding author.

## Ethics statement

The studies involving humans were approved by Taichung Veterans General Hospital Institutional Review Board. The studies were conducted in accordance with the local legislation and institutional requirements. The participants provided their written informed consent to participate in this study. The animal study was approved by Taichung Veterans General Hospital Institutional Review Board. The study was conducted in accordance with the local legislation and institutional requirements.

## Author contributions

SN: Writing – original draft, Writing – review & editing. C-HL: Conceptualization, Data curation, Methodology, Supervision, Writing – review & editing. SH: Conceptualization, Data curation, Writing – review & editing. SH: Conceptualization, Data curation, Writing – review & editing. LS: Conceptualization, Data curation, Writing – review & editing. C-KL: Conceptualization, Data curation, Writing – review & editing. YS: Conceptualization, Data curation, Writing – review & editing. TL: Conceptualization, Data curation, Writing – review & editing. YC: Conceptualization, Data curation, Writing – review & editing. PC: Writing – review & editing, Data curation, Supervision, Conceptualization. LL: Conceptualization, Data curation, Writing – review & editing.
